# Inter- and intra-reader reproducibility of shear wave elastography measurements for musculoskeletal soft tissue masses

**DOI:** 10.1007/s00256-019-03300-2

**Published:** 2019-12-12

**Authors:** Jonathan Nicholls, Abdulrahman M. Alfuraih, Elizabeth M. A. Hensor, Philip Robinson

**Affiliations:** 1 Musculoskeletal Centre X-Ray Department, Leeds Teaching Hospitals Trust, Chapel Allerton Hospital, Chapeltown Road, Leeds, LS7 4SA UK; 2 NIHR Leeds Biomedical Research Centre, Leeds Teaching Hospitals NHS Trust, Leeds, UK; 3 Leeds Institute of Rheumatic and Musculoskeletal Medicine, Chapel Allerton Hospital, University of Leeds, Leeds, UK; 4 Radiology and Medical Imaging Department, College of Applied Medical Sciences, Prince Sattam bin Abdulaziz University, Kharj, Saudi Arabia

**Keywords:** Elastography, Ultrasound, Muscles, Medical imaging, Reliability

## Abstract

**Objective:**

To determine inter- and intra-reader reproducibility of shear wave elastography measurements for musculoskeletal soft tissue masses.

**Materials and methods:**

In all, 64 patients with musculoskeletal soft tissue masses were scanned by two readers prior to biopsy; each taking five measurements of shear wave velocity (m/s) and stiffness (kPa). A single lesion per patient was scanned in transverse and cranio-caudal planes. Depth measurements (cm) and volume (cm^3^) were recorded for each lesion, for each reader. Linear mixed modelling was performed to assess limits of agreement (LOA), inter- and intra-reader repeatability, including analyses for measured depth and volume.

**Results:**

Of the 64 lesions scanned, 24 (38%) were malignant. Bland-Altman plots demonstrated negligible bias with wide LOA for all measurements. Transverse velocity was the most reliable measure—intraclass correlation (95% CI) = 0.917 (0.886, 1)—though reader 1 measures could be between 38% lower and 57% higher than reader 2 [ratio-scale bias (95% LOA) = 0.99 (0.64, 1.55)]. Repeatability coefficients indicated most disagreement resulted from poor within-reader reproducibility. LOA between readers calculated from means of five repeated measurements were narrower—transverse velocity ratio-scale bias (95% LOA) = 1.00 (0.74, 1.35). Depth affected both estimated velocity and repeatability; volume also affected repeatability.

**Conclusion:**

This study found poor repeatability of measurements with wide LOA due mostly to intra-reader variability. Transverse velocity was the most reliable measure; variability may be affected by lesion depth. At least five measurements should be reported with LOA to assist future comparability between shear wave elastography systems in evaluating soft tissue masses.

**Electronic supplementary material:**

The online version of this article (10.1007/s00256-019-03300-2) contains supplementary material, which is available to authorized users.

## Introduction

Shear wave elastography (SWE) offers a novel approach in the investigation of musculoskeletal soft tissue lesions. The technique provides a noninvasive, relatively inexpensive, quantitative measure of local tissue elasticity. To date, only a few feasibility studies have investigated its practical use in delineating benign versus malignant disease, so far showing mixed results [1–4]. Traditional (strain) elastography requires external compression to determine the mechanical properties of tissues [5]. Shear wave elastography utilises modern acoustic radiation force impulse imaging which removes the need for operator compression, theoretically improving repeatability [6, 7].

Shear wave elastography is now a relatively established technique in breast [8, 9], liver [10], and thyroid [11] disease, though remains in the validation phase for musculoskeletal applications [12]. Previous studies have demonstrated moderate to almost perfect SWE repeatability in resting [13–16] and stretched [17, 18] normal skeletal tissue. Multiple previous studies have investigated the diagnostic value of sonoelastography for soft tissue lesions [1, 3, 4, 19, 20]. Despite this, there is only limited published data on the reproducibility and repeatability of SWE and how it may be affected by tumour characteristics such as depth and location. Technical factors have been shown to significantly affect SWE reliability in healthy muscle, including unit of measurement, depth and probe load [14]. To our knowledge, no studies have independently examined reproducibility of shear wave measurements in pathological soft tissue lesions. Furthermore, there is also no data reported on the relatively new SWE system utilised in this study (LOGIQ E9, GE Healthcare, Buckinghamshire, UK) on soft tissue masses, considering that SWE reliability can vary between systems [13, 21]. The aim of this study was to investigate inter- and intra-reader repeatability for shear wave measurements in musculoskeletal soft tissue masses, and to determine the effect of lesion depth and malignant status.

## Material and methods

### Patient population

Ethical approval was obtained from the institutional ethics committee and informed consent acquired from all study participants. Adult patients (*n* = 64) were consecutively referred through local sarcoma services over a 12-month period between March 2016 until March 2017, with no clinical exclusion criteria. The patients in this study were involved in a larger study of imaging characteristics and elastography in benign and malignant masses [22]. Patients were retrospectively selected where five individual elastography readings had been recorded. Malignant status for all extremity lesions was confirmed with histology following soft-tissue biopsy.

### Shear wave elastography imaging

Shear wave elastography was performed on all patients using a single system (LOGIQ E9, GE Healthcare, Buckinghamshire, UK) operating a linear 9–5 MHz probe. This SWE system produces shear waves using the comb-push excitation method that transmits multiple focused acoustic radiation force impulses simultaneously [23–25]. Time-interleaved shear wave tracking is then utilised to measure shear wave velocity, which can also be reported in Young’s elastic modulus [kilopascals (kPa)] using the following equation

$$ E=3\ \rho\ {V}_S^2 $$

where *E* is Young’s modulus, 3 is a constant related to Poisson’s ratio for strain, *ρ* is tissue density (assumed to be 1 g/cm^3^) and *V* is the velocity of shear waves [24]. The system’s software automatically reports this by calculating the sum of the value of each pixel in m/s squared and multiplies it by 3. Both units were reported and documented to two decimal places by the machine and used in the analyses for each unit.

**Fig. 1:**
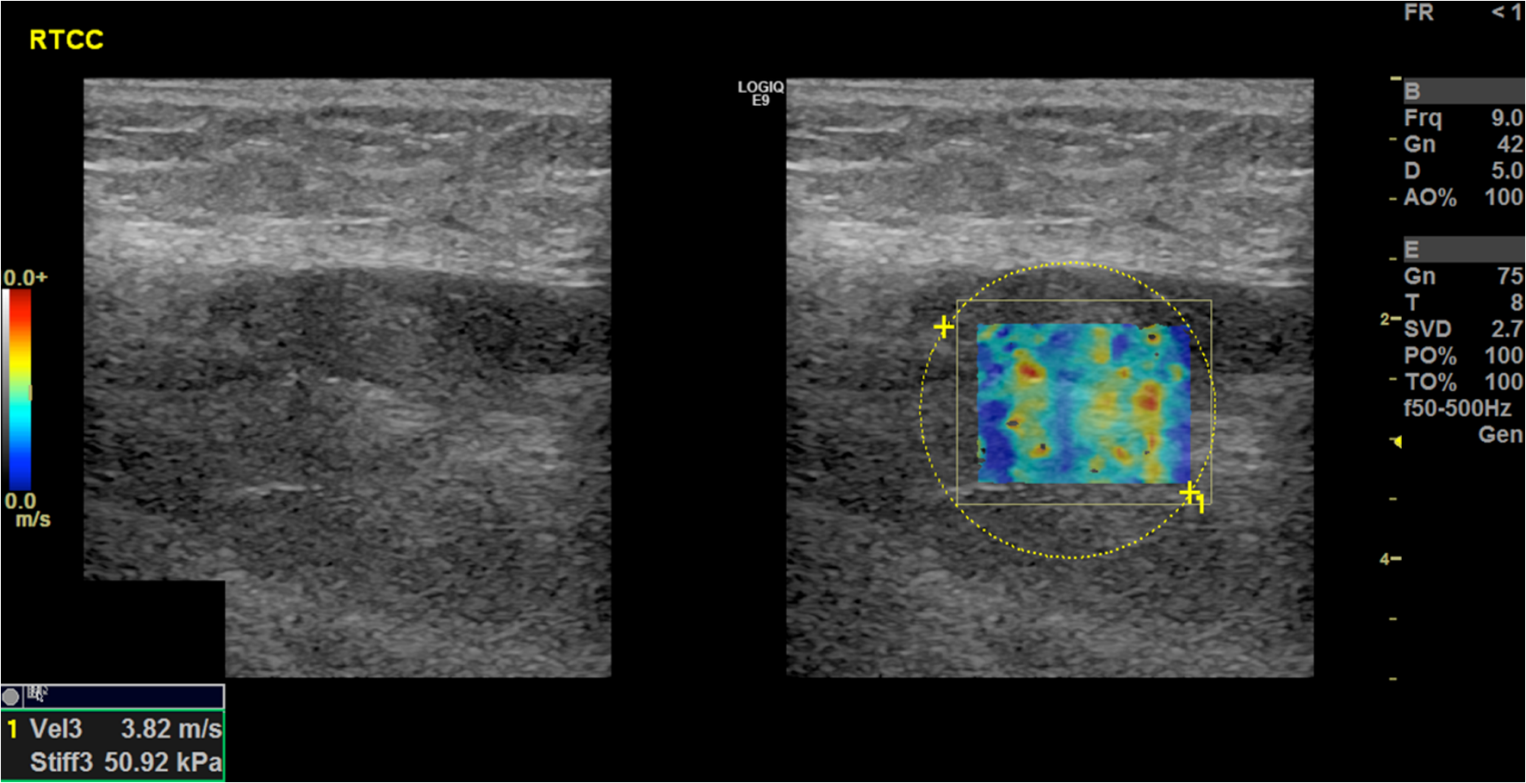
Example of reader defined region of interest modifiable square box located in most superficial aspect of lesion that appeared solid – myxoid liposarcoma

Two experienced readers (blinded to each other) independently scanned a single lesion per patient. Reader 1 (R1) was a board-qualified radiologist (author 4) with >18 years experience (>3 years with SWE). Reader 2 (R2) was a board-qualified sonographer (author 2) with 5 years experience (>2 years with SWE). Patients were placed in relaxed positions depending on the location of the lesion. Positions were adjusted to ensure no active (contraction) or passive (stretching) effects directly influenced the elasticity results. R1 marked the skin surface to determine transducer location and this was also used to place the probe by R2. Each reader made five measurements of shear wave velocity (m/s) and stiffness (kPa) in the transverse and craniocaudal (CC) planes from the most homogenously solid and vascularised region. The same region of the lesion was sampled each time. The probe was lightly replaced between each measurement using minimal probe pressure on the skin surface. Each reader defined the region of interest using a modifiable square box located in the most superficial aspect of the lesion that appeared solid (Fig. 1). Depth of measurement (cm) was recorded for each lesion and for each reader. Lesion volume (cm^3^) was also recorded for each lesion.

### Statistical analysis

To address skewed distributions, all variables were natural log-transformed prior to analysis. Bland-Altman 95% limits of agreement (LOA) and repeatability coefficients, adjusted for repeated measurements, were calculated from variance components of linear mixed models as described by Carstensen et al. [26]. Fixed effects inter- and intra-reader intraclass correlation coefficients (ICCs; single measures definition) were calculated concurrently according to the methods of Eliasziw et al. [27]. In linear mixed models, variance of repeated measurements (level 1), within each patient (level 2), were modelled as a non-linear (quadratic) function of measured lesion depth (cm) and volume (cm^3^), separately for each reader. Analyses were conducted in Stata v14.0 and MLWin v3.0.

## Results

### Shear wave measurements

Of the 64 lesions scanned, 24 (38%) were confirmed to be malignant. Mean patient age was 56 years (range 21 to 90) with 30 males (47%). Mean shear wave measurements by each reader are presented in Table 1.

**Table 1:** Shear wave measurements summarised by reader

Shear wave measure	Reader 1	Reader 2
Transverse velocity, m/s	2.11 (2.01, 2.21)	2.11 (2.01, 2.21)
Transverse stiffness, kPa	15.59 (14.24, 17.07)	15.46 (14.17, 16.88)
Cranio-caudal velocity, m/s	2.14 (2.04, 2.23)	2.02 (1.93, 2.12)
Cranio-caudal stiffness, kPa	15.97 (14.67, 17.39)	14.34 (13.14, 15.65)

All values presented as geometric mean (95% confidence interval)

### Bland-Altman plots

**Fig. 2:**
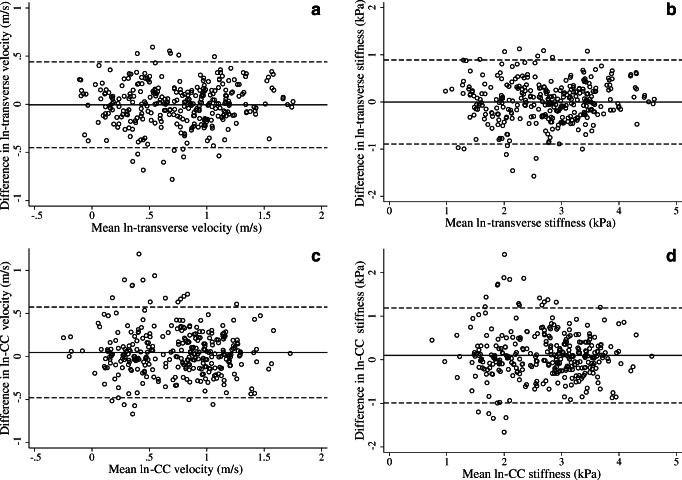
Bland-Altman plots of shear wave measurements on log scale: **a** transverse velocity (m/s); **b** transverse stiffness (kPa); **c** cranio-caudal velocity (m/s); **d** cranio-caudal stiffness (kPa)

Calculation of Bland-Altman mean differences and LOA indicated that mean bias was negligible for transverse measurements. R1 CC measurements tended to be 5–10% higher than R2. For all measures the LOA were wide (Table 2; Fig. 2). For the most reliable measure, transverse velocity (m/s), measurements by R1 could be anywhere between 38% lower and 57% higher than R2. For the least reliable measure, CC stiffness, the measurements could be between 63% lower and 228% higher for R1 compared to R2.

**Table 2:** Repeatability coefficients for each reader and 95% limits of agreement (LOA) between them, adjusted for repeated measures

Shear wave measure	Coefficient of repeatability		Mean difference^a^ (95% LOA)	Mean ratio^b^ (95% LOA)
	Reader 1	Reader 2		
Transverse velocity, m/s	0.34	0.40	−0.01 (−0.45, 0.44)	0.99 (0.64, 1.55)
Transverse stiffness, kPa	0.68	0.82	0.00 (−0.89, 0.89)	1.00 (0.41, 2.44)
Cranio-caudal velocity, m/s	0.35	0.38	0.05 (−0.48, 0.57)	1.05 (0.62, 1.78)
Cranio-caudal stiffness, kPa	0.73	0.76	0.10 (−0.99, 1.19)	1.10 (0.37, 3.28)

*LOA* limits of agreement

^a^Natural log scale

^b^Back-transformed from log scale therefore reported values are ratios between reader 1 and reader 2

### Repeatability coefficients

Repeatability coefficients are defined as the upper 95% prediction limit for the absolute difference between two measurements by the same reader, on the same lesion, under identical circumstances. After comparing repeatability coefficients calculated for each reader to the widths of the LOA calculated on the natural log scale (Table 2), we concluded that most of the disagreement between readers resulted from poor within-reader repeatability.

### Limits of agreement for means of five repeated measurements

Calculating LOA from the means of the five repeated measurements per lesion by each reader resulted in narrower LOA compared to using single measurements (Table 3); nevertheless, transverse velocity measurements by one reader could still be 26% lower or 35% higher than those of the other reader.

**Table 3:** Limits of agreement (LOA) between the two readers calculated using the means of the five repeated measurements

Shear wave measure	Mean difference^a^ (95% LOA)	Mean ratio^b^ (95% LOA)
Transverse velocity, m/s	0.00 (−0.30, 0.30)	1.00 (0.74, 1.35)
Transverse stiffness, kPa	0.01 (−0.58, 0.60)	1.01 (0.56, 1.82)
Cranio-caudal velocity, m/s	0.05 (−0.36, 0.47)	1.06 (0.69, 1.60)
Cranio-caudal stiffness, kPa	0.11 (−0.76, 0.98)	1.11 (0.47, 2.65)

^a^Natural log scale

^b^Back-transformed from log scale therefore reported values are ratios between reader 1 and reader 2

### Intraclass correlation coefficients

Intraclass correlation coefficients for inter- and intra-reader reliability were substantial despite the wide LOA, and also indicated that transverse velocity measurements were the most reliable (Table 4). These results indicate that in populations of similar variability to our sample, shear wave measures, particularly in the transverse plane, can reliably distinguish between patients.

**Table 4:** Fixed inter- and intra-reader intraclass correlation coefficients for each shear wave measure

Shear wave measure	Intraclass correlation coefficient (95% CI^a^)		
	Inter-reader	Intra-reader 1	Intra-reader 2
Transverse velocity, m/s	0.857 (0.811, 1)	0.887 (0.846, 1)	0.917 (0.886, 1)
Transverse stiffness, kPa	0.843 (0.795, 1)	0.872 (0.825, 1)	0.909 (0.875, 1)
Cranio-caudal velocity, m/s	0.786 (0.716, 1)	0.895 (0.851, 1)	0.905 (0.866, 1)
Cranio-caudal stiffness, kPa	0.752 (0.673, 1)	0.882 (0.831, 1)	0.890 (0.843, 1)

^a^One-sided lower limit confidence interval (CI)

### Lesion status

Bland-Altman limits of agreement and intraclass correlation coefficients for transverse shear-wave-velocity measurements are presented by lesion status (benign/malignant) in Tables S1 and S2 of the electronic supplementary material (ESM) respectively; overall agreement and reliability did not differ substantially by status.

### Depth and volume

Both lesion depth and volume may potentially affect repeatability. To explore the impact of depth and volume further we proceeded to model the extent and variability of repeated measurements of transverse velocity as a function of measured lesion depth (cm) and volume (cm^3^). Separate models were constructed for each reader. To avoid over- or underestimating the association between variability and depth by including small numbers of observations at the extremes of the distribution, only lesions located between depths of 0.4 and 5 cm (61/64; 95%), were included in models.

**Fig. 3:**
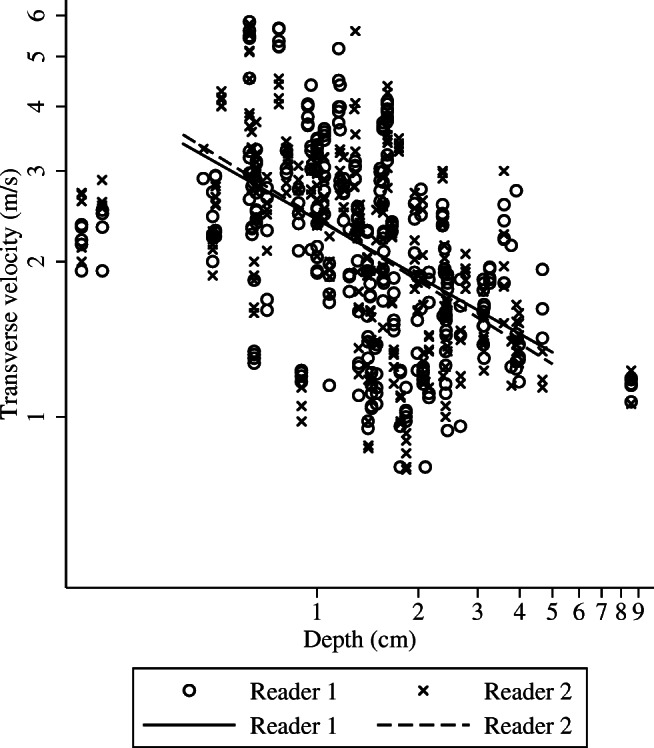
Association between measured lesion depth (cm; log transformed) and transverse shear wave velocity (m/s; log transformed)

The association between depth and measured velocity did not differ significantly between benign and malignant lesions for R1 (*p* = 0.358) or R2 (*p* = 0.261). The same was true for lesion volume (R1: *p* = 0.181; R2: *p* = 101). Combining benign and malignant lesions, on the log scale the difference in velocity per additional cm of depth (standard error**)** was −0.37 (0.08) for R1 (*z* = −4.85, *p* < 0.001) and −0.41 (0.07) for R2 (*z* = −5.88, *p* < 0.001) (Fig. 3). This equates to a decrease in velocity of approximately 3.5% for a 10% increase in depth. There was no evidence that measured velocity varied by depth for either reader (R1: *p* = 0.633; R2: *p* = 0.290).

The variability of shear-wave-velocity measurements in the transverse plane varied to a statistically significant degree according to both lesion depth and volume for both readers (all *p* < 0.01); however the direction of these trends varied between readers. For reader 1, variability increased with depth for lesions of all sizes, but the effect was most pronounced for the largest lesions. For reader 2, variability increased with depth for smaller lesions, but for the larger lesions variability decreased with depth.

## Discussion

Despite several studies investigating the diagnostic role of sonoelastography in musculoskeletal soft tissue masses, none have critically evaluated reproducibility within and between readers in addition to the effect of lesion characteristics. Our study is the first to report on soft tissue masses using the LOGIQ E9 (GE Healthcare, Buckinghamshire, UK). Not all SWE systems can be assumed to be the same, as each manufacturer utilises their own patent-protected technology and variances may exist [13]; therefore, it is important to establish the reliability for each system.

With regard to probe orientation, transverse velocity was the most reliable measure in our data, supporting findings from a previous study regarding benign or malignant soft tissue lesions (*n* = 28) using the Acuson S3000 ultrasound system (Siemens AG, Erlangen, Germany) [1]. The same research group reported slightly higher intra-reader repeatability in the CC orientation on a sample of 50 masses using the Acuson S2000 model (Siemens AG, Erlangen, Germany) [4]. Similarly, studies by Cortez et al. [28] (*n* = 16) and Alfuraih et al. [13] (*n* = 20) reported CC velocity as the most consistent measure in smaller samples of healthy muscle, using the Aixplorer (Supersonic Imagine, Aix-en-Provence, France) and LOGIQ E9 (GE Healthcare, Buckinghamshire, UK) ultrasound systems, respectively. The reason for this is uncertain and may be due to the difference between pathological tissue architecture versus improved longitudinal probe alignment against normal linear muscle fibres [28, 29]. By contrast, a study in breast disease, which has more homogenous tissue with no intervening or surrounding muscle, showed no difference between probe orientations [30]. Despite Bland-Altman plots demonstrating negligible bias, calculated LOAs were wide indicating poor agreement overall. We felt this was predominantly due to poor intra-reader repeatability. Intraclass correlation coefficients for inter- and intra-reader reliability were generally lower than those previously reported in soft tissue masses [1, 4] and healthy muscle [15–18], though values remained substantial (i.e. mostly >0.8). The high intraclass correlation coefficients we obtained indicate that patients from populations with a similar range of values can be reliably distinguished from each other, which may be sufficient for diagnostic purposes. However, discriminative performance would be poorer in less variable populations, and the wide LOA indicate that shear wave measurements may be too variable for evaluative purposes, to monitor a lesion over time for example, unless steps can be taken to reduce the LOA, such as taking the mean of at least 5 repeated measurements.

The high ICCs obtained, despite the wide Bland-Altman LOA, highlight the distinction between reliability (where the aim is to reliably rank patients with different shear wave velocities) and agreement (where the aim is to obtain the same value). If we sought to determine velocity cut-offs indicating whether an individual lesion was likely to be malignant then absolute agreement over the precise shear wave velocity would be important, and the wide LOA would be a concern.

Interestingly, the unit of shear wave velocity (m/s) yielded better results across all outcomes in comparison to stiffness (kPa). This is in agreement with two previous studies that reported a lower reliability for kPa as a result of the mathematical conversion, which depends on pixel averaging meaning that pixel heterogenicity can lead to differing stiffness calculations for the same measured velocity [14, 22]. We therefore support the use of shear wave velocity as a surrogate for tissue elasticity when imaging soft tissue masses as this should ensure better repeatability as evidenced in our results.

No previous studies have investigated the effect of depth (cm) and malignant status on SWE reliability in musculoskeletal soft tissue lesions. Alfuraih et al. [14] recently demonstrated increased SWE variance with depth of measurement in healthy skeletal muscle (*n* = 20). In our study, statistical modelling revealed increased variation at depth in some cases; however, this effect was not observed consistently for both readers. This could be due to the number of repeat measurements made by each reader, which could limit the accuracy of the variance estimates. Yoon et al. found decreased accuracy for both benign and malignant breast lesions at depth (*n* = 47) [31]. Similarly, studies evaluating accuracy and effect of depth in liver assessment show variable reproducibility [32, 33]. The reason for variance at depth in musculoskeletal soft tissue lesions is unclear, though may be explained by increased heterogeneity of deep malignant lesions reducing B-mode image quality, thereby diminishing ability to produce effective push pulses and optimum shear wave tracking [34, 35]. In addition, shear wave propagation through different intervening tissue planes in musculoskeletal lesions may further decrease accuracy. One area of further research would be to investigate the impact of malignancy and lesion position (subcutaneous/deep to fascia) on SWE variance, which would require a larger sample size.

Given wide LOA, we decided to calculate LOA from the mean of five repeated measurements. Alfuraih et al. [13] (*n* = 20) expressed LOA numerically from the mean of three repeated measurements using two different SWE systems. Similarly, Feng at al. [36] (n = 20) displayed LOA graphically from the mean of three repeated measurements using the MyotonPro device (Myoton, Tallinn, Estonia) in normal muscle belly and tendon. Several studies have calculated LOA in nonmusculoskeletal tissues using variable numbers of repeated measurements including liver [37], thyroid [38] and breast [38, 39]—for example, Mulabecirovic et al. [37] found no significant difference using five or ten readings when measuring liver stiffness. Using five measurements in the present study decreased the LOA from (−36%, +55%) to (−26%, +35%); however, we would recommend taking at least five measurements if feasible, to further reduce the LOA.

Although patient numbers were somewhat low (*n* = 64), these were comparatively higher than equivalent studies in soft tissue masses and were statistically adequate for the purposes of our study. In order to meet the assumptions of the linear mixed models and the Bland-Altman limits of agreement, shear wave variables were log-transformed. This approach was recommended by Bland and Altman [40] to address issues of associations between the extent and variability of measurement and should not hinder interpretation, as results can subsequently be back-transformed and expressed as ratios. Using nonlinear (polynomial) terms to model level 1 variance, although a recommended method, can lead to unreliable predictions at the extremes of the distribution; therefore, the wide divergence between predicted variance of the two readers at the greatest depths should be interpreted with caution [41]. However, over the range of observed values, the results suggest that variance was associated with lesion depth and volume, and the association differed qualitatively between the two readers.

## Conclusion

In summary, of the four readings available (CC, transverse, m/s, kPa), transverse shear wave velocity (m/s) was the most reliable measure in musculoskeletal soft tissue masses. Despite limited evidence of bias and substantial inter- and intra-reader intraclass correlation coefficient values, LOA were wide which was most likely due to poor intra-reader repeatability. We recommend future reliability studies use the mean of at least five repeated measurements and provide LOA to improve ease of comparability between SWE systems as technologies evolve. Authors should include lesion depth and volume in musculoskeletal soft tissue masses as these factors are likely to compound intra-reader LOA.

## Electronic supplementary material

ESM 1 (DOCX 27 kb)
