# Supplementary material for: Inter- and intra-reader reproducibility of shear wave elastography measurements for musculoskeletal soft tissue masses
Source: Skeletal Radiol. 2019 Dec 12;49(5):779–86. doi: 10.1007/s00256-019-03300-2 (PMC7083807; doi:10.1007/s00256-019-03300-2)
Supplement: Supplementary file 1 — (DOCX 27 kb) [file 256_2019_3300_MOESM1_ESM.docx]

### Supplementary Material

### Table S1: Repeatability coefficients for shear wave velocity measurements in the transverse plane by each reader and 95% limits of agreement (LOA) between them, adjusted for repeated measures, by lesion status

| Lesion status | Coefficient of repeatability | | Mean difference* (95% LOA) | Mean ratio** (95% LOA) |
| --- | --- | --- | --- | --- |
|  | **Reader 1** | **Reader 2** |  |  |
| Benign | 0.35 | 0.38 | -0.02 (-0.47, 0.44) | 0.99 (0.62, 1.56) |
| Malignant | 0.32 | 0.43 | 0.01 (-0.42, 0.44) | 1.01 (0.66, 1.55) |

*LOA=Limits of agreement *Natural log scale **Back-transformed from log scale therefore reported values are ratios between reader 1 and reader 2*

### Table S2: Fixed inter- and intra-reader intraclass correlation coefficients for shear wave velocity measurements in the transverse plane, by lesion status

| Lesion status | Intraclass correlation coefficient (95% CI*) | | |
| --- | --- | --- | --- |
|  | **Inter-reader** | **Intra-reader 1** | **Intra-reader 2** |
| Benign | .863 (.805, 1) | .907 (.862, 1) | .925 (.889, 1) |
| Malignant | .850 (.770, 1) | .853 (.769, 1) | .915 (.863, 1) |

**One-sided lower limit confidence interval (CI)*
